# Computation of the kinematics and the minimum peak joint moments of sit-to-stand movements

**DOI:** 10.1186/1475-925X-6-26

**Published:** 2007-07-03

**Authors:** Shinsuke Yoshioka, Akinori Nagano, Ryutaro Himeno, Senshi Fukashiro

**Affiliations:** 1 Department of Life Sciences (Sports Sciences), University of Tokyo, Japan; 2 Computational Biomechanics Unit, Riken, Japan; 3 Institute of Medical Sciences, University of Aberdeen, UK; 4 Graduate School of Interdisciplinary Information Studies, University of Tokyo, Japan

## Abstract

**Background:**

A sit-to-stand (STS) movement requires muscle strength higher than that of other daily activities. There are many elderly people, who experience difficulty when standing up from a chair. The muscle strength required (or the load on the joints) during a STS task is determined by the kinematics (movement pattern). The purpose of this study was to evaluate the kinematics and resultant joint moments of people standing up from a chair in order to determine the minimum peak joint moments required for a STS task.

**Methods:**

This study consisted of three steps. In the first step, kinematic data of lower extremity joint angles (hip, knee and ankle) during STS movements were experimentally collected from human subjects. Eighty-five sets of STS kinematic data were obtained. In the second step, the experimentally collected kinematic data and a link segment model of the human body were used to generate more than 5,000,000 computed STS movements. In the third step, using inverse dynamics method, joint moments of the lower extremity were calculated for all movements obtained through the preceding steps. From the outputs of the third step, the optimal kinematics (movement pattern) in terms of minimized peak joint moment for the hip, knee and ankle was determined.

**Results:**

The peak hip joint moment ranged from 0.24 to 1.92 N.m/kg. The peak knee joint moment ranged from 0.51 to 1.97 N.m/kg, and the peak ankle joint moment ranged from -0.11 to 1.32 N.m/kg. The optimal movement patterns differed depending on which minimized joint moment index was selected (hip, knee or ankle). However, the sum of the peak hip joint moment and peak knee joint moment was always approximately 1.53 N.m/kg regardless of which minimized joint moment index was selected.

**Conclusion:**

The most important finding of this study was that the relation between the peak joint moments at the hip and knee joints was complementary and the sum of those moments needed to be greater than 1.53 N.m/kg in order to perform a successful STS. A combined hip-knee value of 1.5 N.m/kg or lower may indicate the need for physical rehabilitation and/or exercise to increase muscular force.

## Background

A sit-to-stand (STS) movement, which is defined as a movement of standing up from a chair to an upright posture, is one of the most demanding daily activities in mechanical terms. A STS movement requires a peak joint moment greater than other movements such as stair climbing or walking [1], and yields peak hip joint contact pressure higher than other movements such as walking, jogging or jumping [2]. Hodge et al. (1989) used a specially built hip endoprosthesis equipped with pressure measuring transducers. They showed that the peak hip contact pressure between the acetabulum of the pelvis and the femoral head during a STS movement was greater than that during walking, jogging or jumping. Also, a STS movement requires muscle strength greater than other daily activities, such as walking or stair climbing [3]. Additionally, there are many elderly people, who experience difficulty when standing up from a chair [4,5]. Such difficulties influence the quality of daily life and ability to remain independent. Therefore, the STS task has been studied in many preceding research projects with a strong focus on the muscle strength. For example, Hughes et al. showed that the peak value of the knee joint moment during STS movements reached up to 97% of the maximum isometric knee extensor strength when the chair height was low [6]. They suggested that the knee extensor strength was the limiting factor of the STS movement from a low chair height. Rantanen et al. showed that there was a significant difference in the normalized isometric strengths of five muscle groups (hand grip, elbow flexion, knee extension, trunk extension and trunk flexion) between the subjects who could stand up from a chair without difficulty and the subjects who had difficulties when standing up from a chair [7]. These are valuable findings that have contributed to the biomechanical understanding of human daily activities.

However, muscle strength is not the only determinant of STS performance. Schlicht et al. reported that STS performance did not improve despite an increase of muscle strength through an intense strength training program [8]. Schultz et al. compared the joint moments during STS movement with published values of the maximum voluntary muscle strength and showed that the joint moments at the lift off were well below the literature values with an exception of nursing home residents [9]. In addition, they investigated the location of the ground reaction force as the index of postural stability and showed that there were clear differences among three groups (young, elderly without STS difficulty and elderly with STS difficulty). From those findings, they suggested that the postural stability at the lift off was the major determinant of STS ability. Whitney et al. reported that the five-times-sit-to-stand test [10] score of the subjects with balance disorders were lower than that of the subjects without balance disorders [11]. Additionally, Lord et al. showed that, using multiple regression analysis, not only muscle strength and balancing ability but also other physiological (sensorimotor condition) and psychological (pain, depression, anxiety and vitality) factors influenced STS performance [12]. Therefore, to understand a STS movement more thoroughly and utilize the findings of STS studies for therapy, therapeutic intervention programs and evaluation of STS performance, it is important that STS movements are studied from various perspectives.

A STS movement pattern (kinematics) is generated as a result of mechanical actions of the muscles, which are controlled by central neural commands. Although, it is assumed that there is a biomechanically "optimal" movement pattern of STS depending on the intention (such as to stand up quickly, easily, safely, etc.), there is a possibility that an individual is not always conducting optimal STS movements. In other words, it might be possible to enhance the STS performance by changing the currently employed movement pattern of each person to an optimal one. Therefore, it is important to study the optimality of STS movement patterns (kinematics). If the kinematics are properly adjusted, the kinetics may be changed requiring less muscle force and joint moments, even if the chair height, body mass or body height are identical. There is a possibility that it takes less time to achieve kinematic improvements than improving muscle strength or balancing ability. Therefore, it is useful for therapists, doctors or patients to understand the kinematics in which people can stand up with low joint moments, and to know the minimum peak joint moment required for a STS task.

The purpose of this study was to reveal the kinematics with which people can stand up from a chair with the minimum peak joint moment, and to evaluate the minimum peak joint moment required for a STS task.

## Methods

This study consisted of three steps. In the first step, kinematic data of lower extremity joint angles (hip, knee and ankle) during STS movements were experimentally collected. Eighty-five sets of STS kinematic data were obtained from human subjects. In the second step, using those kinematic data and a link segment model of the human body, more than 5,000,000 movements were computationally generated. In the third step, for all movements obtained through the preceding steps, the joint moments of the lower extremity were calculated using an inverse dynamics method. From the outputs of the third step, the kinematics that produced the minimum peak joint moments were determined. Throughout this study, bilateral symmetry was assumed.

### 1) Joint kinematic data collection

Five healthy young male subjects (age 26 ± 3 years, height 1.74 ± 0.04 m, mass 73.8 ± 3.4 kg) participated in this experiment with informed consent. None of them had any known musculoskeletal or neurological disorders. This project was performed under the approval of the ethics committee of the University of Tokyo.

**Figure 1:**
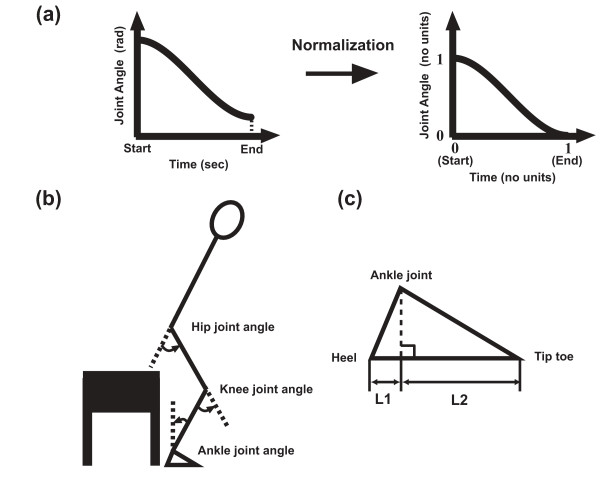
**The joint kinematics normalization, joint definition and the foot segment definition**. (a) The translation process from the raw movement data to the normalized format suitable for the computer model. (b) Hip, knee and ankle joint definitions. A counterclockwise angle has a positive value. (c) Details of the foot segment size. L1 indicates the horizontal length between the heel and the ankle joint. L2 indicates the horizontal length between the ankle joint and the tip toe.

To obtain lower extremity joint kinematics during STS movements, 3D coordinates of the landmark points of the subject's body were acquired using a 3D optical motion capture system with 7 cameras at 200 Hz (Hawk Digital System, Motion Analysis Corporation, Santa Rosa, CA, USA). Seven reflective markers were placed on the subject's body (the right acrominon, sacroiliac joint, right and left anterior superior iliac spines, right epicondylus lateralis, right malleolus lateralis and the distal end of the fifth metatarsal). All raw coordinates data were smoothed using a fourth-order butterworth low-pass digital filter. The cut off frequency (7 Hz) was determined with a residual analysis [13]. The hip joint center position was calculated from the sacroiliac joint, right and left anterior superior iliac spines and right epicondylus lateralis [14]. Joint angles were calculated from those coordinate data. The joint angles were defined as shown in Fig. 1b. The chair height was set at 0.40 m, since the Japan Industrial Standard and British Standards Institute recommend 0.40 m as the standard chair height (JIS S 1011 and JIS S 1015) and the toilet pedestal height [15], respectively.

The subject's arms were folded across the chest. The subjects wore corsets for the neck (COLLAR KEEPER me, Nippon Sigmax, Tokyo, Japan) and back (MAXBELT CH, Nippon Sigmax, Tokyo, Japan) to prevent the head-arm-trunk (HAT) segment from bending except at the hip joints. (The HAT segment was assumed to be a rigid body.) STS movement was initiated from a squat posture in which the subject's buttocks lightly touched the chair. In this study, joint moment development during the STS task was the focus of analysis. In the sitting phase, since the body is supported by the chair, the load imposed on the lower limb is small. In preceding studies, Schenkman et al. (1990), Kotake et al. (1993) and Kralj et al. (1990) reported that the joint moments reach the maximum after the buttocks lose contact with the chair [16-18]. Therefore, the STS movement was simplified and only the rising phase was analyzed. This design was appropriate for the purpose of this study. Each subject was instructed to perform a total of 50 STS movements using various speeds and movement patterns without countermovement or arm support. The initial posture and feet position of the subjects were not restricted. The movements in which a countermovement was generated were excluded from further analyses. A joint movement greater than 3 degrees in the opposite direction was regarded as a countermovement and rejected. Since the number of successful trials that were compliant with the instruction ranged between 17 and 44 among the subjects, 17 trials per subject were used for further analysis (for those subjects who had performed a greater number of successful trials, 17 trials were randomly selected). The number of the trials for each subject was restricted to 17 to avoid the possibility that the kinematics of certain subjects influence the final results more than others. As a result, 85 trials (5 subjects, 17 trials per subject) were adopted in total. To translate the raw movement data into a format suitable for use in the next step, the joint angle time series data were normalized about the movement time and the range of change in the joint angle between the initial posture and the standing posture (Fig. 1a). The start and finish time were determined based on the joint angle deviation (3 deg) with respect to the stationary initial and final joint angles, respectively. The normalized data were fitted with 8th-order polynomial equations that produced average residual error from the experimental data of less than 1%. This fitting was used in order to adjust the time scale of hip, knee and ankle joint kinematic data at the next computation step.

### 2) Computation of STS movements

The planar link segment model of the human body developed for this study consisted of four segments (HAT, thigh, shank and foot segments) and three joints (hip, knee and ankle joints). A segment was connected to the adjacent segment(s) with a frictionless uniaxial hinge joint. It was assumed that, no slip or rotation occurred between the bottom of the foot segment and the surface of the floor. To obtain the body segmental parameter values for this model, human anthropometric data [13,19] were scaled to fit the average height and mass (1.74 m, 73.8 kg) of the experimental subjects. The foot segment lengths L1 (0.0454 m) and L2 (0.2018 m) were obtained by averaging the measurement values from the subjects (Fig. 1c). Each lower extremity joint angle was defined as shown in Fig. 1b. The joint angles were defined as 0 deg when this model was standing upright. The height of the hip joint center of this model at the initial posture was set at the average height (0.513 m) of the experimental movements, in order to make the initial hip joint height of the experimental and computed STS movements identical. The joint kinematic data acquired from the preceding steps were used as the input parameters of this link segment model.

**Figure 2:**
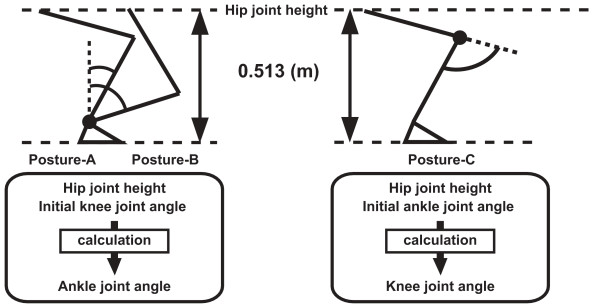
**The adjustment process for the parameters influencing the initial posture**. The adjustment process for the three parameters influencing the initial posture (hip joint height, initial knee joint angle and initial ankle joint angle). Initial ankle joint angles in Posture-A and Posture-B were calculated from the hip joint height and initial knee joint angle. In Posture-C, initial knee joint angle was calculated from the hip joint height and initial ankle joint angle.

The model had three joints and each joint had 85 variations of joint kinematic data. Therefore, the total combination of joint kinematics was 614,125 (85 × 85 × 85). As the original experimental movements of the three joint kinematics were independent, two inconsistencies occurred in the model. The first inconsistency was related to adjusting the kinematic data of each joint with respect to the movement time so that a movement began and finished in a coordinated manner. For this purpose, three manipulations were performed, since the three joint kinematic data had three different movement times. First, the knee and ankle movement time data were adjusted to the movement time of the hip. Second, the hip and ankle movement time data were adjusted to the movement time of the knee. Finally, the hip and knee movement time data were adjusted to the movement time of the ankle. Therefore, three movements of different durations were acquired from one set of hip, knee and ankle joint kinematic data. The second inconsistency was related to matching the hip joint height of the computed movement to the average hip joint height (0.513 m) of the experimental movements at the initial posture. The initial hip joint height of the computed movement is determined by the ankle and knee initial joint angles. As the initial hip joint height was set as 0.513 m, the initial knee joint angle of the linked segment model is determined as a function of the initial ankle joint angle, or vice versa (the initial ankle joint angle is determined as a function of the initial knee joint angle). However, in the experimental data, the initial knee and ankle joint angles were independent, which caused the second inconsistency. To resolve this issue, when the ankle (knee) initial joint angle was used to determine the initial posture of the computed movement, the knee (ankle) joint angle was adjusted. As a result, three initial postures were derived from one set of hip, knee and ankle joint kinematic data (Fig. 2). Posture-A and Posture-B were derived from the initial joint angle of the knee joint kinematic data, while Posture-C was derived from that of the ankle joint kinematic data. The movement time (× 3) and initial hip joint height adjustments (× 3) resulted in 9 movement patterns being acquired for each set of the combined joint kinematic data. As a result, in this step, more than 5,000,000 (9 × 85 × 85 × 85) variations of STS movements were acquired from the 85 variations of joint kinematic data and the link segment model.

### 3) Joint moment calculation

For each STS movement, the coordinates of the center of pressure (COP) on the floor and the joint moments of each lower extremity joint were calculated using an inverse dynamics method (equations of motion are shown in Appendix). Inverse dynamic calculation was applied from the HAT segment toward the foot segment with the motion data and the human body segmental parameters reported in preceding studies [13,19]. When the horizontal speed of STS movement is zero and there is no external force (except for the gravity force and the ground reaction force), if the location of the COP does not stay within the foot support range at the moment of rising from the seat, the STS movement cannot be completed. The STS movements investigated in this study were assumed to be slow, since this study focused on the minimum joint moment requirement. Therefore, the movements in which the coordinates of the COP did not stay within the model's foot support range were assumed to be unsuccessful, and were excluded from further analysis. The peak joint moment of each lower extremity joint during each movement was identified. Joint moments of hip extension, knee extension and ankle plantar flexion were defined as positive. According to the model's dynamic equations, the values of joint moment changes linearly with the model's mass. Therefore, the joint moments were normalized by the model's mass (73.8 kg).

The joint moment requirement of each successful computed trial was evaluated using four joint moment indices (|M_H_|, |M_K_|, |M_A_| and |M_H _+ M_K _+ M_A_|). M_H_, M_K _and M_A _are the peak joint moment at the hip, knee and ankle joints, respectively (|M_H_|, |M_K_| and |M_A_| are the absolute values). A negative joint moment value means an opposite direction of force exertion. Therefore, the absolute values of the peak joint moments were used as joint moment indices. The joint moment indices |M_H_|, |M_K_| and |M_A_|, were used to determine the optimal movement, i.e. the movement in which a minimized peak movement is developed for the specified joint. These indices may be useful for helping to correct STS strategies in people whose hip, knee or ankle joint moment development ability is relatively weak compared to the other two joints. The |M_H _+ M_K _+ M_A_| index was used to determine the movement in which the peak moments of all three joints were minimized, and can therefore be thought of as the optimal "overall" kinematic strategy. This index could be used to help improve STS performance in people who have equally low moment development ability in each lower limb joint.

## Results

**Figure 3:**
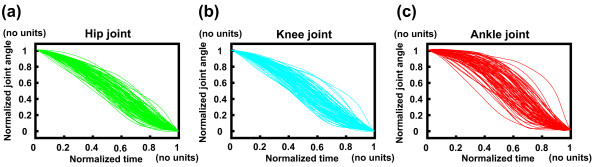
**Normalized joint angle time series data**. Normalized hip (a), knee (b), and ankle (c) joint angle time series data obtained from the experimental trials (n = 17 × 5).

**Figure 4:**
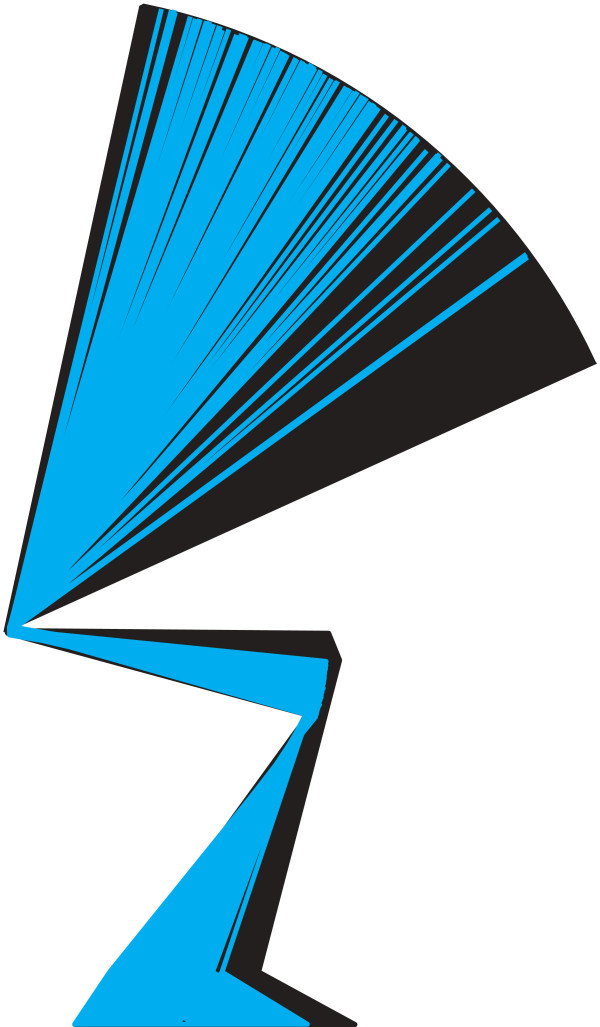
**Initial postures of the experimental and computed movements**. Stick figures of the initial postures of the experimental and computed movements drawn with a fixed hip joint center position. Blue figures are the initial postures of the experimental movements. Black figures are the initial postures of the computed movements.

**Figure 5:**
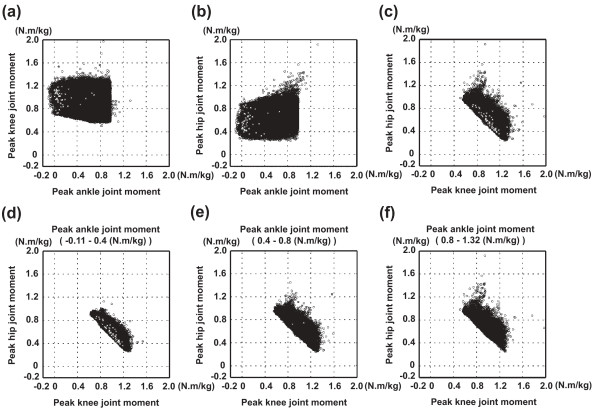
**Peak joint moments of each movement**. (Top row) The relation between the peak joint moments of each movement. Plotted data include: the relation between peak joint moments at the ankle and knee joints (a); the relation between peak joint moments at the ankle and hip joints (b); and the relation between peak joint moments at the knee and hip joints (c). (Bottom row) The relation between peak knee and hip joint moments with 3 different ranges ankle joint moments. The peak ankle joint moments ranged from -0.11 to 0.4 N.m/kg (d); 0.4 to 0.8 N.m/kg (e); and 0.8 to 1.32 N.m/kg (f). The plots ((d), (e) and (f)) show the relation between the peak ankle joint moment and other two joint moments. Plots (d), (e), and (f) all show similar distribution profiles. The value of the peak ankle joint moment did not influence the distribution pattern of other two joint moments. Joint moments are the values for one leg. The cross mark plots indicated the data of the original experimental data.

Eighty-five normalized joint angle time series data per joint were experimentally obtained (Fig. 3). There were large variations of joint angle kinematic data and a wide range of total movement time (0.34 to 14.72 s) data, which provided the necessary inputs for the computer simulation model. Of the 5,527,125 movement patterns (9 × 85 × 85 × 85) that were generated, 160,086 movements were adopted as successful. The remaining unsuccessful movements were rejected since the location of the COP did not stay within the model's foot support range during the movement. The initial postures of the experimental and computed movements are shown in Fig. 4. The ankle, knee and hip joint angles of the successfully computed movements ranged between (-39 and -19 deg), (102 and 114 deg) and (-138 and -89 deg), respectively. In the experimental movements, those angles ranged between (-39 and -19 deg), (102 and 113 deg) and (-138 and -89 deg), respectively. Therefore, the ranges of those angles of the successfully computed movements were similar to those of the experimental movements. This suggests that most (or at least, many) of the successful movements were within the range of experimental observation and were not unrealistic movements. The peak joint moments of each simulation were identified and plotted in Fig. 5. The peak hip joint moment ranged from 0.24 to 1.92 N.m/kg. The peak knee joint moment ranged from 0.51 to 1.97 N.m/kg, and the peak ankle joint moment ranged from -0.11 to 1.32 N.m/kg. The profile of the distribution pattern of (c) differed from the profile of either (a) or (b). The same data were plotted from a different perspective in Fig. 5 (bottom row). The plots (Fig. 5(d), (e) and 5(f)) display the relation between the peak ankle joint moment and other two joint moments. Plots (d), (e), and (f) all show similar distribution profiles. This implies that the value of the peak ankle joint moment did not influence the distribution pattern of other two joint moments.

**Figure 6:**
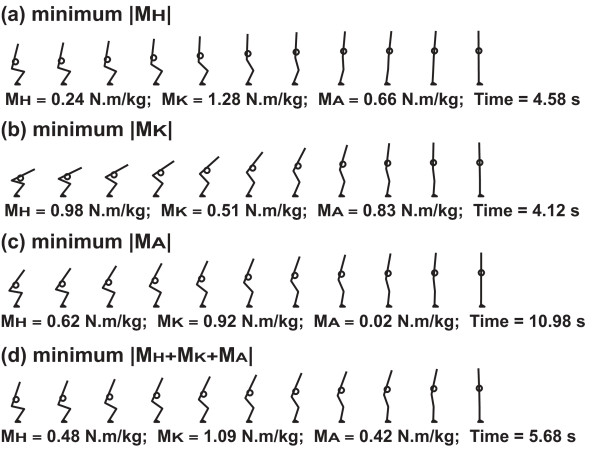
**Movement pattern, peak joint moments, and movement time**. Movement pattern, peak joint moments, and movement time for each joint moment index. M_H_, M_K _and M_A _are peak joint moment at the hip, knee and ankle joints during the movement, respectively. |M_H_|, |M_K_| and |M_A_| are the absolute values. The index value of each movement was the smallest among the values of all computed movements. Circle mark indicates the total body center of gravity.

**Figure 7:**
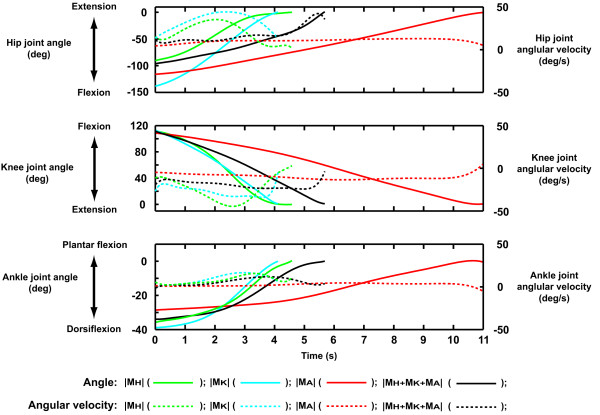
**The profile of the joint angles and angular velocities**. Time = 0 indicates the instance when the buttocks lost contact with the chair. All graphs are drawn to the end of each STS movement. The left and right axes are the scale of the joint angle and angular velocity, respectively. The solid and dotted lines show the joint angle and angular velocity of each joint, respectively.

The movement patterns in which the joint moment index value was the minimum were shown in Fig. 6. The sum of the peak hip joint moment and peak knee joint moment were always approximately 1.53 N.m/kg in these movement patterns. That is, when the peak hip joint moment was minimum (0.24 N.m/kg), the peak knee joint moment increased accordingly (1.28 N.m/kg) (Fig. 6(a)). The resultant posture of this movement was characterized by a relatively vertical HAT segment throughout. Similarly, when the peak knee joint moment was minimized (0.51 N.m/kg), the peak hip joint moment increased to 0.98 N.m/kg (Fig. 6(b)). This movement pattern resulted in a HAT segment tilted in the forward direction at the initiation of the movement. The movement patterns in which |M_A_| or |M_H _+ M_K _+ M_A_| were minimized had intermediate characteristics between the movement patterns in which |M_H_| or |M_K_| was minimized. The smallest value (-0.11 N.m/kg) of the peak ankle joint moment was a negative value (Fig. 6). In this case, a dorsiflexion moment was exerted throughout the entire movement sequence. The hip, knee and ankle joint angles during the STS movement, derived according to each joint moment index, are shown in Fig. 7.

**Figure 8:**
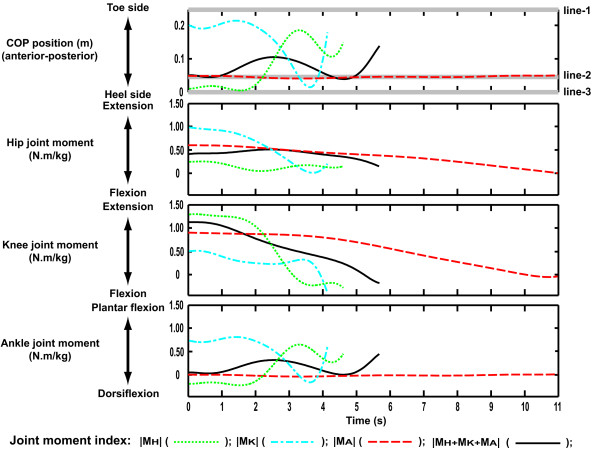
**The profile of the anterior-posterior COP position and joint moments**. Time = 0 indicates the instance when the buttocks lost contact with the chair. All graphs are drawn until the end of each STS movement. The upper end of the graph indicates tip toe position (line-1). The lower limit of the same graph indicates the heel position (line-3). The intermediate line indicates ankle joint position (line-2). Joint moments are the values for one leg. Peak hip and knee joint moments reached their maximum near the initial position. At the ankle, joint moments tended to increase or decrease in synchrony with the changes in the anterior-posterior position of the COP. At the early stage of the movement, the hip joint moment exhibited the highest value when the joint moment index was |M_K_|. The magnitude of the hip joint moments decreased in order from |M_A_|, |M_H _+ M_K _+ M_A_| and |M_H_|, respectively. In the case of the knee joint moment, the order was the opposite.

The anterior-posterior position of the COP, and the hip, knee and ankle joint moments during the STS movement, derived according to each joint moment index, are shown in Fig. 8. Peak hip and knee joint moments reached their maximum near the initial position. At the ankle, joint moments were generally lower and tended to increase or decrease in synchrony with the changes in the anterior-posterior position of the COP. At the early stage of the movement, the hip joint moment exhibited the highest value when the joint moment index was |M_K_|. The magnitude of the hip joint moments decreased in order from |M_A_|, |M_H _+ M_K _+ M_A_| and |M_H_|, respectively. In the case of the knee joint moment, the order was the opposite. A complementary relation between the hip and knee joints was observed.

## Discussion

The purpose of this study was to reveal the kinematics (movement pattern) with which people can stand up from a chair with minimum peak joint moment (Fig. 6 and Fig. 7), and to quantitatively evaluate the minimum peak joint moment required for a STS task. Each row in Fig. 6 represents a minimized joint moment index. For a model with a stature of 1.74 m to successfully stand up from a chair height of 0.40 m, the minimum peak joint moments required at the hip, knee and ankle joint were 0.24, 0.51 and 0.02 N.m/kg, respectively. The minimum value for the sum of peak hip joint moment and peak knee joint moment was approximately 1.53 N.m/kg. The data in Fig. 5 (bottom row) and Fig. 6 show that the relation between the peak joint moments at the hip and knee joints was complementary. Even for different magnitudes of peak ankle joint moment, the distribution patterns of all graphs were similar to each other (Fig. 5(d), (e) and 5(f)). It was also observed that the ankle joint moment was closely related to the anterior-posterior position of the COP rather than the hip or knee joint moment (Fig. 8). Therefore, it was found that, while the hip and knee peak joint moments were related to each other, peak ankle joint moment was not related to either hip or knee peak joint moment.

There was a clear variation in the optimal movement patterns according to the indices of joint moment requirement (Fig. 6). When the index was |M_H_|, |M_K_| or |M_A_|, the total body center of gravity (CG) was located above the hip, knee or ankle joints during the movements, respectively (Fig. 6(a), (b) and 6(c)). However, when the index was |M_H _+ M_K _+ M_A_|, the total body CG was not maintained above a certain point, but the horizontal trajectory of the total body CG was similar to that of the anterior-posterior position of the COP (Fig. 8). The inclination of the HAT segment plays an important role in the adjustment of the location of the total body CG. This suggests that, for example, if the moment development ability of the knee joint is relatively weak, it may be better to stand up from a chair with a movement pattern in which the HAT segment is tilted in the forward direction so that the total body CG is maintained above the knee joint as Fig. 6(b).

The movement strategy of the |M_H _+ M_K _+ M_A_| index had intermediate characteristics between those of the |M_H_| and |M_K_| indices (Fig. 6(a), (b) and 6(d)). The peak hip, knee and ankle joint moments of the movement of the |M_H _+ M_K _+ M_A_| index were higher than the minimum peak hip, knee and ankle joint moments of all the computed movements. However, the peak joint moments at the three joints were evenly low. For example, in the case of the |M_H_| index, though the hip joint moment of the movement of the |M_H_| index was lower than that of the movement of the |M_H _+ M_K _+ M_A_| index, the other two joint moments were higher. Therefore, for people being in a state of more general weakness at all three joints, it might be better to recommend the movement strategy of the |M_H _+ M_K _+ M_A_| index (Fig. 6(d)). The movement strategy of the |M_A_| index was similar to that of the |M_H _+ M_K _+ M_A_| index. In the case of the movement of the |M_A_| index, the moment output at the ankle joint during the movement was nearly zero. Also, there were many successful movements of which peak ankle joint moments were nearly zero (Fig. 5). Therefore, the ankle joint moment is not necessarily required to complete the STS task. However, there were no successful movements in which the peak hip or knee joint moment was nearly zero. In other words, a certain magnitude of hip and knee joint moment development is necessary to complete the STS task. Therefore, it is suggested that the role of the ankle joint moment during the STS movement is different from that of the hip or knee joint moment. Also, there is a possibility that people automatically optimize their STS strategies according to their moment development abilities. It might be possible to predict the moment development ability of a subject based on his/her movement strategy. Actually, there were some experimental movements similar to the movements shown in Fig. 6. This will be an interesting future research theme.

As shown in Fig. 6, the sum of peak hip joint moment and peak knee joint moment was relatively invariant throughout the range of movement patterns. On the other hand, each of the peak joint moments was greatly affected by the movement pattern. Similar trends have been shown in previous studies. Winter (1980) assessed the kinetics during the stance phase of gait and reported that the time-series sum of lower extremity joint moments remained relatively consistent despite variations within individual joints [20]. It was concluded that, to assess gait kinetics, the sum of lower extremity joint moments should be examined, since the analysis for single joint kinetics might lead to an erroneous diagnosis. The STS movement and the stance phase of gait have similar characteristics, as both movements require weight bearing and propulsion of the center of mass [21]. Shepherd and Gentile showed that the peak values for the sum of lower extremity joint moments during STS movement were similar despite variations of the peak moment at each joint. Our study has shown that the minimum sum of the hip and knee moments is relatively invariant over a range of standing strategies. Therefore, based on the findings of our study and those previous studies, it is reasonable to emphasize the biomechanical importance of the sum of the minimum peak joint moments at the hip and knee joints over isolated analyses of each joint.

Cahalan et al., and Markhede and Grimby reported the isokinetic peak hip extensor moments (60–90 deg/s) of community dwellers [22,23]. Those values ranged between approximately 70 and 220 N.m. Larsson et al. and Runnels et al. performed a study on the knee joint moment outputs [24,25]. The isokinetic knee extensor moments (60 deg/s) ranged between approximately 90 and 230 N.m. Both hip and knee extensor moments of community dwellers were greater than the minimum peak joint moments obtained in this study. On the other hand, the isokinetic knee extensor moment of nursing home residents (25.8 N.m, 60 deg/s) reported by Whipple et al. was lower than the minimum peak joint moments obtained in this study [26]. These comparisons support the reliability of the results of this study. However, usually one research result can not be directly compared with another, as there could be differences in the experimental condition, and the peak joint moment is affected by many factors such as the subject's physical properties and experimental condition. Therefore, these comparisons have to be made with caution.

In Fig. 5(c), the knee and hip peak joint moments were distributed in the lower right area of the graph rather than in the upper left area. In the lower right area of the graph, the value of peak knee joint moment was higher than that of peak hip joint moment. In preceding studies, the moment development ability of the knee joint (90 – 230 N.m) [24,25] has been reported to be similar to that of the hip joint (70 – 220 N.m) [22,23]. Therefore, it is suggested that the moment development ability of the knee joint is a more influential limiting factor of the STS movement than that of the hip joint. This insight is in accordance with the suggestions of previous studies [27,28].

The movement patterns that exhibited optimal joint moment characteristics for each of the indices were all slow (Fig. 6). The movement time ranged between 4.12 and 10.98 s. These movement times seem to be relatively longer than the movement times of normal STS movements. When the movement time is long, there is a possibility that the STS movement is affected by the ability to maintain the muscle force exertion due to fatigue. Therefore, it is necessary to consider the slowness of the movements. The joint moments can be divided into static and inertial components [29]. The speed of the movement only influences the inertial component. Hughes et al. (1996) examined the contribution of the inertial component of the knee joint moment during an STS task and showed that if the movement time is greater than 1.5 s, the contribution of the inertial component could be ignored [27]. Therefore, it is assumed that, if the joint trajectories of two STS movements are the same and both the movement times are greater than 1.5 s, the joint moments, COP locations and ground reaction forces of the movements are almost the same. Therefore when researchers, doctors and therapists consider the movements of which the movement times are about 2 or 3 seconds, they can apply the findings of this study (i.e., minimum peak joint moments required for STS movement and movement strategies) to those movements. It seems likely that the slowness of the movements has no practical influence on the results and does not matter for general clinical applications.

In this study, the optimal movements for each joint moment index were determined from computed movements derived from a wide range of actual human trials. To obtain such a wide range of human trial data, it was important to use subjects physically capable of performing said movements. It was assumed that, due to limited strength in elderly populations, young subjects would be better able to accomplish the STS task with various movement strategies (from normal to extreme strategies). Therefore, young males were used as the subject of this study.

Fig. 4 shows all of the initial postures of the experimental and computed movements. From Fig. 4, it was assumed that the range of the kinematic data acquired from the experiment was enough for the purpose of this study. Also, Ikeda et al. (1991) and Linden et al. (1994) showed that the movement pattern of the elderly was similar to that of the young [30,31]. Alexander et al. (1991) showed that the time and angle data of males and females were comparable [4]. Kerr et al. (1997) analyzed 50 normal subjects of varying ages and both sexes. They showed that the results of the analysis supported a general consistency across both sexes and all age groups except movement time and forward lean of the trunk. In the movement time and forward lean of the trunk, there were differences across both sexes and all age groups. However, in this study, a wide range of movements (movement time (0.34 to 14.72 s) and forward lean of the trunk (Fig. 4)) were computed and analyzed. Therefore, it is assumed that the results of this study could be applicable to the elderly and young women as well, although the results of this study were based on the motion data acquired from young male subjects. It would be valuable to perform detailed comparisons between different age groups and sexes in future studies.

Restrictions of the neck and back with the corsets were imposed to prevent the head-arm-trunk (HAT) segment from bending except at the hip joints. The possibility that these restrictions may have lead to unrealistic movement strategies can not be excluded. However, unrealistic movements were not observed in the experimental phase of this study. Additionally, the computed results of this study (for example. Fig. 6) seemed to be realistic. Therefore, it was considered that these restrictions did not lead to unrealistic movement strategies.

The minimum peak joint moments obtained in this study indicate the joint moment thresholds for standing up from a chair. However, there are many other factors (balancing ability, sensorimotor condition, pain, depression, anxiety and vitality) that limit STS performance. Even if a person is able to develop joint moments that exceed the minimum peak joint moments obtained in this study, it does not automatically mean that s/he will have the ability to stand up from a chair. That is to say other factors might be hindering STS performance.

In this study, to focus on the joint moment of the lower extremity, the contribution of the upper extremity use was not taken into consideration. In daily life, there are many cases in which people have to stand up from a chair without upper extremity support, for example, when they are carrying objects in both hands. Therefore, it was assumed that the usefulness of this study is not reduced by this simplification. Empirically, it has been recognized that using the upper extremity can increase the ease of the STS movement. Also, researchers have studied the contribution of the upper extremity from the viewpoint of muscle strength[32,33] or balancing ability [4,9]. While the use of the arms was restricted in this study, we acknowledge that future studies should consider the contribution of the upper extremity during STS movements.

## Conclusion

We examined large variations of STS movement patterns to determine the minimum peak joint moments required to perform a STS task. Four optimal movement patterns (kinematics) for four joint moment indices were revealed. Those four movement patterns differed mainly in the HAT segment inclination during the movements. Therefore, when doctors and therapists need to find out the optimal movement pattern for each person from the viewpoint of muscle strength, it may be important to focus on HAT segment inclination. The minimum peak joint moments required for STS task were also revealed. The values at the hip, knee and ankle were 0.24, 0.51 and 0.02 N.m/kg, respectively. Additionally, it was revealed that the relation between the peak joint moments at the hip and knee joints was complementary and the sum of those moments needed to be greater than 1.53 N.m/kg. A combination hip-knee value of 1.5~N.m/kg may be an indication that physical rehabilitation and exercise prescription for the improvement of muscular force development is necessary to complete the STS task. This value may be useful for doctors, therapists and a variety of populations with reduced strength and/or mobility, since the relation of the hip and knee joints is clear.

## Competing interests

The author(s) declare that they have no competing interests.

## Authors' contributions

SY performed the data collection and analyses, constructed the simulation model and drafted the manuscript. AN participated in the process of data collection, analysis, model construction and manuscript writing. RH and SF contributed discussions and suggestions throughout this project, including the manuscript writing. All authors read and approved the final manuscript.

## Appendix

### 1) Equations of motion

The following equations of motion were used to calculate the joint moments. In this study, bilateral symmetry was assumed.

(HAT segment)

mHAT⋅d2dt2P→HAT_CM=mHAT⋅g→+2⋅F→HAT_Hip
 MathType@MTEF@5@5@+=feaafiart1ev1aaatCvAUfKttLearuWrP9MDH5MBPbIqV92AaeXatLxBI9gBaebbnrfifHhDYfgasaacH8akY=wiFfYdH8Gipec8Eeeu0xXdbba9frFj0=OqFfea0dXdd9vqai=hGuQ8kuc9pgc9s8qqaq=dirpe0xb9q8qiLsFr0=vr0=vr0dc8meaabaqaciaacaGaaeqabaqabeGadaaakeaacqWGTbqBdaWgaaWcbaGaemisaGKaemyqaeKaemivaqfabeaakiabgwSixpaalaaabaGaemizaq2aaWbaaSqabeaacqaIYaGmaaaakeaacqWGKbazcqWG0baDdaahaaWcbeqaaiabikdaYaaaaaGcdaWhcaqaaiabdcfaqbGaay51GaWaaSbaaSqaaiabdIeaijabdgeabjabdsfaujabc+faFjabdoeadjabd2eanbqabaGccqGH9aqpcqWGTbqBdaWgaaWcbaGaemisaGKaemyqaeKaemivaqfabeaakiabgwSixlqbdEgaNzaalaGaey4kaSIaeGOmaiJaeyyXIC9aa8HaaeaacqWGgbGraiaawEniamaaBaaaleaacqWGibascqWGbbqqcqWGubavcqGGFbWxcqWGibascqWGPbqAcqWGWbaCaeqaaaaa@5D6B@

ddt(IHAT_CM⋅ω→HAT_CM)=2⋅(R→HATCM_Hip×F→HAT_Hip+M→HAT_Hip)
 MathType@MTEF@5@5@+=feaafiart1ev1aaatCvAUfKttLearuWrP9MDH5MBPbIqV92AaeXatLxBI9gBaebbnrfifHhDYfgasaacH8akY=wiFfYdH8Gipec8Eeeu0xXdbba9frFj0=OqFfea0dXdd9vqai=hGuQ8kuc9pgc9s8qqaq=dirpe0xb9q8qiLsFr0=vr0=vr0dc8meaabaqaciaacaGaaeqabaqabeGadaaakeaadaWcaaqaaiabdsgaKbqaaiabdsgaKjabdsha0baacqGGOaakcqWGjbqsdaWgaaWcbaGaemisaGKaemyqaeKaemivaqLaei4xa8Laem4qamKaemyta0eabeaakiabgwSixJGaciqb=L8a3zaalaWaaSbaaSqaaiabdIeaijabdgeabjabdsfaujabc+faFjabdoeadjabd2eanbqabaGccqGGPaqkcqGH9aqpcqaIYaGmcqGHflY1cqGGOaakdaWhcaqaaiabdkfasbGaay51GaWaaSbaaSqaaiabdIeaijabdgeabjabdsfaujabdoeadjabd2eanjabc+faFjabdIeaijabdMgaPjabdchaWbqabaGccqGHxdaTdaWhcaqaaiabdAeagbGaay51GaWaaSbaaSqaaiabdIeaijabdgeabjabdsfaujabc+faFjabdIeaijabdMgaPjabdchaWbqabaGccqGHRaWkdaWhcaqaaiabd2eanbGaay51GaWaaSbaaSqaaiabdIeaijabdgeabjabdsfaujabc+faFjabdIeaijabdMgaPjabdchaWbqabaGccqGGPaqkaaa@737C@

(Thigh segment)

mThigh⋅d2dt2P→Thigh_CM=mThigh⋅g→+F→Thigh_Hip+F→Thigh_Knee
 MathType@MTEF@5@5@+=feaafiart1ev1aaatCvAUfKttLearuWrP9MDH5MBPbIqV92AaeXatLxBI9gBaebbnrfifHhDYfgasaacH8akY=wiFfYdH8Gipec8Eeeu0xXdbba9frFj0=OqFfea0dXdd9vqai=hGuQ8kuc9pgc9s8qqaq=dirpe0xb9q8qiLsFr0=vr0=vr0dc8meaabaqaciaacaGaaeqabaqabeGadaaakeaacqWGTbqBdaWgaaWcbaGaemivaqLaemiAaGMaemyAaKMaem4zaCMaemiAaGgabeaakiabgwSixpaalaaabaGaemizaq2aaWbaaSqabeaacqaIYaGmaaaakeaacqWGKbazcqWG0baDdaahaaWcbeqaaiabikdaYaaaaaGcdaWhcaqaaiabdcfaqbGaay51GaWaaSbaaSqaaiabdsfaujabdIgaOjabdMgaPjabdEgaNjabdIgaOjabc+faFjabdoeadjabd2eanbqabaGccqGH9aqpcqWGTbqBdaWgaaWcbaGaemivaqLaemiAaGMaemyAaKMaem4zaCMaemiAaGgabeaakiabgwSixpaaFiaabaGaem4zaCgacaGLxdcacqGHRaWkdaWhcaqaaiabdAeagbGaay51GaWaaSbaaSqaaiabdsfaujabdIgaOjabdMgaPjabdEgaNjabdIgaOjabc+faFjabdIeaijabdMgaPjabdchaWbqabaGccqGHRaWkdaWhcaqaaiabdAeagbGaay51GaWaaSbaaSqaaiabdsfaujabdIgaOjabdMgaPjabdEgaNjabdIgaOjabc+faFjabdUealjabd6gaUjabdwgaLjabdwgaLbqabaaaaa@79B7@

ddt(IThigh_CM⋅ω→Thigh_CM)=R→ThighCM_Hip×F→Thigh_Hip+R→ThighCM_Knee×F→Thigh_Knee+M→Thigh_Hip+M→Thigh_Knee
 MathType@MTEF@5@5@+=feaafiart1ev1aaatCvAUfKttLearuWrP9MDH5MBPbIqV92AaeXatLxBI9gBaebbnrfifHhDYfgasaacH8akY=wiFfYdH8Gipec8Eeeu0xXdbba9frFj0=OqFfea0dXdd9vqai=hGuQ8kuc9pgc9s8qqaq=dirpe0xb9q8qiLsFr0=vr0=vr0dc8meaabaqaciaacaGaaeqabaqabeGadaaakeaafaqaaeGabaaabaWaaSaaaeaacqWGKbazaeaacqWGKbazcqWG0baDaaGaeiikaGIaemysaK0aaSbaaSqaaiabdsfaujabdIgaOjabdMgaPjabdEgaNjabdIgaOjabc+faFjabdoeadjabd2eanbqabaGccqGHflY1daWhcaqaaGGaciab=L8a3bGaay51GaWaaSbaaSqaaiabdsfaujabdIgaOjabdMgaPjabdEgaNjabdIgaOjabc+faFjabdoeadjabd2eanbqabaGccqGGPaqkcqGH9aqpaeaadaWhcaqaaiabdkfasbGaay51GaWaaSbaaSqaaiabdsfaujabdIgaOjabdMgaPjabdEgaNjabdIgaOjabdoeadjabd2eanjabc+faFjabdIeaijabdMgaPjabdchaWbqabaGccqGHxdaTdaWhcaqaaiabdAeagbGaay51GaWaaSbaaSqaaiabdsfaujabdIgaOjabdMgaPjabdEgaNjabdIgaOjabc+faFjabdIeaijabdMgaPjabdchaWbqabaGccqGHRaWkdaWhcaqaaiabdkfasbGaay51GaWaaSbaaSqaaiabdsfaujabdIgaOjabdMgaPjabdEgaNjabdIgaOjabdoeadjabd2eanjabc+faFjabdUealjabd6gaUjabdwgaLjabdwgaLbqabaGccqGHxdaTdaWhcaqaaiabdAeagbGaay51GaWaaSbaaSqaaiabdsfaujabdIgaOjabdMgaPjabdEgaNjabdIgaOjabc+faFjabdUealjabd6gaUjabdwgaLjabdwgaLbqabaGccqGHRaWkdaWhcaqaaiabd2eanbGaay51GaWaaSbaaSqaaiabdsfaujabdIgaOjabdMgaPjabdEgaNjabdIgaOjabc+faFjabdIeaijabdMgaPjabdchaWbqabaGccqGHRaWkdaWhcaqaaiabd2eanbGaay51GaWaaSbaaSqaaiabdsfaujabdIgaOjabdMgaPjabdEgaNjabdIgaOjabc+faFjabdUealjabd6gaUjabdwgaLjabdwgaLbqabaaaaaaa@B6B1@

(Shank segment)

mShank⋅d2dt2P→Shank_CM=mShank⋅g→+F→Shank_Knee+F→Shank_Ankle
 MathType@MTEF@5@5@+=feaafiart1ev1aaatCvAUfKttLearuWrP9MDH5MBPbIqV92AaeXatLxBI9gBaebbnrfifHhDYfgasaacH8akY=wiFfYdH8Gipec8Eeeu0xXdbba9frFj0=OqFfea0dXdd9vqai=hGuQ8kuc9pgc9s8qqaq=dirpe0xb9q8qiLsFr0=vr0=vr0dc8meaabaqaciaacaGaaeqabaqabeGadaaakeaacqWGTbqBdaWgaaWcbaGaem4uamLaemiAaGMaemyyaeMaemOBa4Maem4AaSgabeaakiabgwSixpaalaaabaGaemizaq2aaWbaaSqabeaacqaIYaGmaaaakeaacqWGKbazcqWG0baDdaahaaWcbeqaaiabikdaYaaaaaGcdaWhcaqaaiabdcfaqbGaay51GaWaaSbaaSqaaiabdofatjabdIgaOjabdggaHjabd6gaUjabdUgaRjabc+faFjabdoeadjabd2eanbqabaGccqGH9aqpcqWGTbqBdaWgaaWcbaGaem4uamLaemiAaGMaemyyaeMaemOBa4Maem4AaSgabeaakiabgwSixpaaFiaabaGaem4zaCgacaGLxdcacqGHRaWkdaWhcaqaaiabdAeagbGaay51GaWaaSbaaSqaaiabdofatjabdIgaOjabdggaHjabd6gaUjabdUgaRjabc+faFjabdUealjabd6gaUjabdwgaLjabdwgaLbqabaGccqGHRaWkdaWhcaqaaiabdAeagbGaay51GaWaaSbaaSqaaiabdofatjabdIgaOjabdggaHjabd6gaUjabdUgaRjabc+faFjabdgeabjabd6gaUjabdUgaRjabdYgaSjabdwgaLbqabaaaaa@7C67@

ddt(IShank_CM⋅ω→Shank_CM)=R→ShankCM_Knee×F→Shank_Knee+R→ShankCM_Ankle×F→Shank_Ankle+M→Shank_Knee+M→Shank_Ankle
 MathType@MTEF@5@5@+=feaafiart1ev1aaatCvAUfKttLearuWrP9MDH5MBPbIqV92AaeXatLxBI9gBaebbnrfifHhDYfgasaacH8akY=wiFfYdH8Gipec8Eeeu0xXdbba9frFj0=OqFfea0dXdd9vqai=hGuQ8kuc9pgc9s8qqaq=dirpe0xb9q8qiLsFr0=vr0=vr0dc8meaabaqaciaacaGaaeqabaqabeGadaaakeaafaqaaeGabaaabaWaaSaaaeaacqWGKbazaeaacqWGKbazcqWG0baDaaGaeiikaGIaemysaK0aaSbaaSqaaiabdofatjabdIgaOjabdggaHjabd6gaUjabdUgaRjabc+faFjabdoeadjabd2eanbqabaGccqGHflY1daWhcaqaaGGaciab=L8a3bGaay51GaWaaSbaaSqaaiabdofatjabdIgaOjabdggaHjabd6gaUjabdUgaRjabc+faFjabdoeadjabd2eanbqabaGccqGGPaqkcqGH9aqpaeaadaWhcaqaaiabdkfasbGaay51GaWaaSbaaSqaaiabdofatjabdIgaOjabdggaHjabd6gaUjabdUgaRjabdoeadjabd2eanjabc+faFjabdUealjabd6gaUjabdwgaLjabdwgaLbqabaGccqGHxdaTdaWhcaqaaiabdAeagbGaay51GaWaaSbaaSqaaiabdofatjabdIgaOjabdggaHjabd6gaUjabdUgaRjabc+faFjabdUealjabd6gaUjabdwgaLjabdwgaLbqabaGccqGHRaWkdaWhcaqaaiabdkfasbGaay51GaWaaSbaaSqaaiabdofatjabdIgaOjabdggaHjabd6gaUjabdUgaRjabdoeadjabd2eanjabc+faFjabdgeabjabd6gaUjabdUgaRjabdYgaSjabdwgaLbqabaGccqGHxdaTdaWhcaqaaiabdAeagbGaay51GaWaaSbaaSqaaiabdofatjabdIgaOjabdggaHjabd6gaUjabdUgaRjabc+faFjabdgeabjabd6gaUjabdUgaRjabdYgaSjabdwgaLbqabaGccqGHRaWkdaWhcaqaaiabd2eanbGaay51GaWaaSbaaSqaaiabdofatjabdIgaOjabdggaHjabd6gaUjabdUgaRjabc+faFjabdUealjabd6gaUjabdwgaLjabdwgaLbqabaGccqGHRaWkdaWhcaqaaiabd2eanbGaay51GaWaaSbaaSqaaiabdofatjabdIgaOjabdggaHjabd6gaUjabdUgaRjabc+faFjabdgeabjabd6gaUjabdUgaRjabdYgaSjabdwgaLbqabaaaaaaa@BEB3@

(Foot segment)

mFoot⋅d2dt2P→Foot_CM=mFoot⋅g→+F→Foot_Ankle+F→Foot_GRF
 MathType@MTEF@5@5@+=feaafiart1ev1aaatCvAUfKttLearuWrP9MDH5MBPbIqV92AaeXatLxBI9gBaebbnrfifHhDYfgasaacH8akY=wiFfYdH8Gipec8Eeeu0xXdbba9frFj0=OqFfea0dXdd9vqai=hGuQ8kuc9pgc9s8qqaq=dirpe0xb9q8qiLsFr0=vr0=vr0dc8meaabaqaciaacaGaaeqabaqabeGadaaakeaacqWGTbqBdaWgaaWcbaGaemOrayKaem4Ba8Maem4Ba8MaemiDaqhabeaakiabgwSixpaalaaabaGaemizaq2aaWbaaSqabeaacqaIYaGmaaaakeaacqWGKbazcqWG0baDdaahaaWcbeqaaiabikdaYaaaaaGcdaWhcaqaaiabdcfaqbGaay51GaWaaSbaaSqaaiabdAeagjabd+gaVjabd+gaVjabdsha0jabc+faFjabdoeadjabd2eanbqabaGccqGH9aqpcqWGTbqBdaWgaaWcbaGaemOrayKaem4Ba8Maem4Ba8MaemiDaqhabeaakiabgwSixpaaFiaabaGaem4zaCgacaGLxdcacqGHRaWkdaWhcaqaaiabdAeagbGaay51GaWaaSbaaSqaaiabdAeagjabd+gaVjabd+gaVjabdsha0jabc+faFjabdgeabjabd6gaUjabdUgaRjabdYgaSjabdwgaLbqabaGccqGHRaWkdaWhcaqaaiabdAeagbGaay51GaWaaSbaaSqaaiabdAeagjabd+gaVjabd+gaVjabdsha0jabc+faFjabdEeahjabdkfasjabdAeagbqabaaaaa@7447@

ddt(IFoot_CM⋅ω→Foot_CM)=R→FootCM_Ankle×F→Foot_Ankle+R→FootCM_COP×F→Foot_GRF+M→Foot_Ankle
 MathType@MTEF@5@5@+=feaafiart1ev1aaatCvAUfKttLearuWrP9MDH5MBPbIqV92AaeXatLxBI9gBaebbnrfifHhDYfgasaacH8akY=wiFfYdH8Gipec8Eeeu0xXdbba9frFj0=OqFfea0dXdd9vqai=hGuQ8kuc9pgc9s8qqaq=dirpe0xb9q8qiLsFr0=vr0=vr0dc8meaabaqaciaacaGaaeqabaqabeGadaaakeaafaqaaeWabaaabaWaaSaaaeaacqWGKbazaeaacqWGKbazcqWG0baDaaGaeiikaGIaemysaK0aaSbaaSqaaiabdAeagjabd+gaVjabd+gaVjabdsha0jabc+faFjabdoeadjabd2eanbqabaGccqGHflY1daWhcaqaaGGaciab=L8a3bGaay51GaWaaSbaaSqaaiabdAeagjabd+gaVjabd+gaVjabdsha0jabc+faFjabdoeadjabd2eanbqabaGccqGGPaqkcqGH9aqpaeaadaWhcaqaaiabdkfasbGaay51GaWaaSbaaSqaaiabdAeagjabd+gaVjabd+gaVjabdsha0jabdoeadjabd2eanjabc+faFjabdgeabjabd6gaUjabdUgaRjabdYgaSjabdwgaLbqabaGccqGHxdaTdaWhcaqaaiabdAeagbGaay51GaWaaSbaaSqaaiabdAeagjabd+gaVjabd+gaVjabdsha0jabc+faFjabdgeabjabd6gaUjabdUgaRjabdYgaSjabdwgaLbqabaGccqGHRaWkdaWhcaqaaiabdkfasbGaay51GaWaaSbaaSqaaiabdAeagjabd+gaVjabd+gaVjabdsha0jabdoeadjabd2eanjabc+faFjabdoeadjabd+eapjabdcfaqbqabaGccqGHxdaTdaWhcaqaaiabdAeagbGaay51GaWaaSbaaSqaaiabdAeagjabd+gaVjabd+gaVjabdsha0jabc+faFjabdEeahjabdkfasjabdAeagbqabaaakeaacqGHRaWkdaWhcaqaaiabd2eanbGaay51GaWaaSbaaSqaaiabdAeagjabd+gaVjabd+gaVjabdsha0jabc+faFjabdgeabjabd6gaUjabdUgaRjabdYgaSjabdwgaLbqabaaaaaaa@A14E@

d2dt2P→Foot_CM=0
 MathType@MTEF@5@5@+=feaafiart1ev1aaatCvAUfKttLearuWrP9MDH5MBPbIqV92AaeXatLxBI9gBaebbnrfifHhDYfgasaacH8akY=wiFfYdH8Gipec8Eeeu0xXdbba9frFj0=OqFfea0dXdd9vqai=hGuQ8kuc9pgc9s8qqaq=dirpe0xb9q8qiLsFr0=vr0=vr0dc8meaabaqaciaacaGaaeqabaqabeGadaaakeaadaWcaaqaaiabdsgaKnaaCaaaleqabaGaeGOmaidaaaGcbaGaemizaqMaemiDaq3aaWbaaSqabeaacqaIYaGmaaaaaOWaa8HaaeaacqWGqbauaiaawEniamaaBaaaleaacqWGgbGrcqWGVbWBcqWGVbWBcqWG0baDcqGGFbWxcqWGdbWqcqWGnbqtaeqaaOGaeyypa0JaeGimaadaaa@40F4@

ω→Foot_CM=0
 MathType@MTEF@5@5@+=feaafiart1ev1aaatCvAUfKttLearuWrP9MDH5MBPbIqV92AaeXatLxBI9gBaebbnrfifHhDYfgasaacH8akY=wiFfYdH8Gipec8Eeeu0xXdbba9frFj0=OqFfea0dXdd9vqai=hGuQ8kuc9pgc9s8qqaq=dirpe0xb9q8qiLsFr0=vr0=vr0dc8meaabaqaciaacaGaaeqabaqabeGadaaakeaadaWhcaqaaGGaciab=L8a3bGaay51GaWaaSbaaSqaaiabdAeagjabd+gaVjabd+gaVjabdsha0jabc+faFjabdoeadjabd2eanbqabaGccqGH9aqpcqaIWaamaaa@3B2A@

### 2) Nomenclature

*m*_*i *_mass of segment *i*

g→
 MathType@MTEF@5@5@+=feaafiart1ev1aaatCvAUfKttLearuWrP9MDH5MBPbIqV92AaeXatLxBI9gBaebbnrfifHhDYfgasaacH8akY=wiFfYdH8Gipec8Eeeu0xXdbba9frFj0=OqFfea0dXdd9vqai=hGuQ8kuc9pgc9s8qqaq=dirpe0xb9q8qiLsFr0=vr0=vr0dc8meaabaqaciaacaGaaeqabaqabeGadaaakeaadaWhcaqaaiabdEgaNbGaay51Gaaaaa@2FB7@ acceleration of gravity

P→i_CM
 MathType@MTEF@5@5@+=feaafiart1ev1aaatCvAUfKttLearuWrP9MDH5MBPbIqV92AaeXatLxBI9gBaebbnrfifHhDYfgasaacH8akY=wiFfYdH8Gipec8Eeeu0xXdbba9frFj0=OqFfea0dXdd9vqai=hGuQ8kuc9pgc9s8qqaq=dirpe0xb9q8qiLsFr0=vr0=vr0dc8meaabaqaciaacaGaaeqabaqabeGadaaakeaadaWhcaqaaiabdcfaqbGaay51GaWaaSbaaSqaaiabdMgaPjabc+faFjabdoeadjabd2eanbqabaaaaa@3488@ position of the center of mass of segment *i*

*I*_*i_CM *_moment of inertia of segment *i *about the center of mass

ω→i_CM
 MathType@MTEF@5@5@+=feaafiart1ev1aaatCvAUfKttLearuWrP9MDH5MBPbIqV92AaeXatLxBI9gBaebbnrfifHhDYfgasaacH8akY=wiFfYdH8Gipec8Eeeu0xXdbba9frFj0=OqFfea0dXdd9vqai=hGuQ8kuc9pgc9s8qqaq=dirpe0xb9q8qiLsFr0=vr0=vr0dc8meaabaqaciaacaGaaeqabaqabeGadaaakeaadaWhcaqaaGGaciab=L8a3bGaay51GaWaaSbaaSqaaiabdMgaPjabc+faFjabdoeadjabd2eanbqabaaaaa@3533@ angular velocity of segment *i *about the center of mass

R→A_B
 MathType@MTEF@5@5@+=feaafiart1ev1aaatCvAUfKttLearuWrP9MDH5MBPbIqV92AaeXatLxBI9gBaebbnrfifHhDYfgasaacH8akY=wiFfYdH8Gipec8Eeeu0xXdbba9frFj0=OqFfea0dXdd9vqai=hGuQ8kuc9pgc9s8qqaq=dirpe0xb9q8qiLsFr0=vr0=vr0dc8meaabaqaciaacaGaaeqabaqabeGadaaakeaadaWhcaqaaiabdkfasbGaay51GaWaaSbaaSqaaiabdgeabjabc+faFjabdkeacbqabaaaaa@3317@ position vector drawn from point A to point B

F→i_C
 MathType@MTEF@5@5@+=feaafiart1ev1aaatCvAUfKttLearuWrP9MDH5MBPbIqV92AaeXatLxBI9gBaebbnrfifHhDYfgasaacH8akY=wiFfYdH8Gipec8Eeeu0xXdbba9frFj0=OqFfea0dXdd9vqai=hGuQ8kuc9pgc9s8qqaq=dirpe0xb9q8qiLsFr0=vr0=vr0dc8meaabaqaciaacaGaaeqabaqabeGadaaakeaadaWhcaqaaiabdAeagbGaay51GaWaaSbaaSqaaiabdMgaPjabc+faFjabdoeadbqabaaaaa@3351@ force acting on point C of segment *i*

M→i_C
 MathType@MTEF@5@5@+=feaafiart1ev1aaatCvAUfKttLearuWrP9MDH5MBPbIqV92AaeXatLxBI9gBaebbnrfifHhDYfgasaacH8akY=wiFfYdH8Gipec8Eeeu0xXdbba9frFj0=OqFfea0dXdd9vqai=hGuQ8kuc9pgc9s8qqaq=dirpe0xb9q8qiLsFr0=vr0=vr0dc8meaabaqaciaacaGaaeqabaqabeGadaaakeaadaWhcaqaaiabd2eanbGaay51GaWaaSbaaSqaaiabdMgaPjabc+faFjabdoeadbqabaaaaa@335F@ moment acting on segment *i*

COP center of pressure of Foot segment

GRF ground reaction force acting on Foot segment
